# Dynamic cerebral autoregulation after intracerebral hemorrhage: A case-control study

**DOI:** 10.1186/1471-2377-11-108

**Published:** 2011-08-31

**Authors:** Kazuma Nakagawa, Jorge M Serrador, Sarah L LaRose, Farzaneh A Sorond

**Affiliations:** 1 Neuroscience Institute, The Queen's Medical Center, Honolulu, Hawaii, USA; 2 Department of Medicine, John A. Burns School of Medicine, University of Hawaii, Honolulu, Hawaii, USA; 3 War Related Injury and Illness Study Center, New Jersey Veterans Administration & Veterans Biomedical Research Institute, East Orange, New Jersey, USA; 4 Department of Neurology, Brigham and Women's Hospital, Harvard Medical School, Boston, Massachusetts, USA

**Keywords:** Cerebral autoregulation, Intracerebral hemorrhage, TCD Ultrasound

## Abstract

**Background:**

Dynamic cerebral autoregulation after intracerebral hemorrhage (ICH) remains poorly understood. We performed a case-control study to compare dynamic autoregulation between ICH patients and healthy controls.

**Methods:**

Twenty-one patients (66 ± 15 years) with early (< 72 hours) lobar or basal ganglia ICH were prospectively studied and compared to twenty-three age-matched controls (65 ± 9 years). Continuous measures of mean flow velocity (MFV) in the middle cerebral artery and mean arterial blood pressure (MAP) were obtained over 5 min. Cerebrovascular resistance index (CVR_i_) was calculated as the ratio of MAP to MFV. Dynamic cerebral autoregulation was assessed using transfer function analysis of spontaneous MAP and MFV oscillations in the low (0.03-0.15 Hz) and high (0.15-0.5 Hz) frequency ranges.

**Results:**

The ICH group demonstrated higher CVR_i _compared to controls (ipsilateral: 1.91 ± 1.01 mmHg·s·cm^-1^, *p *= 0.04; contralateral: 2.01 ± 1.24 mmHg·s·cm^-1^, *p *= 0.04; vs. control: 1.42 ± 0.45 mmHg·s·cm^-1^). The ICH group had higher gains than controls in the low (ipsilateral: 1.33 ± 0.58%/mmHg, *p *= 0.0005; contralateral: 1.47 ± 0.98%/mmHg, *p *= 0.004; vs. control: 0.82 ± 0.30%/mmHg) and high (ipsilateral: 2.11 ± 1.31%/mmHg, *p *< 0.0001; contralateral: 2.14 ± 1.49%/mmHg, *p *< 0.0001; vs. control: 0.66 ± 0.26%/mmHg) frequency ranges. The ICH group also had higher coherence in the contralateral hemisphere than the control (ICH contralateral: 0.53 ± 0.38, *p *= 0.02; vs. control: 0.38 ± 0.15) in the high frequency range.

**Conclusions:**

Patients with ICH had higher gains in a wide range of frequency ranges compared to controls. These findings suggest that dynamic cerebral autoregulation may be less effective in the early days after ICH. Further study is needed to determine the relationship between hematoma size and severity of autoregulation impairment.

## Background

Cerebral autoregulation serves as a protective feedback mechanism to maintain relatively constant blood flow to the brain in response to changes in cerebral perfusion pressure (CPP) [1]. However, cerebrovascular injuries can impair cerebral autoregulation [2-4] and may result in undesirable ischemia or hyperemia with changes in CPP. Recent studies have demonstrated the importance of impaired cerebral autoregulation as a potential contributor to secondary neuronal damage after traumatic brain injury (TBI) [5], subarachnoid hemorrhage (SAH) [6], ischemic stroke [7] and intracerebral hemorrhage (ICH) [8]. With increasing emphasis on assessing cerebral autoregulation as an important physiologic variable, an emerging concept called "autoregulation-oriented therapy" is beginning to focus on individually adjusting the CPP to compensate for the cerebral autoregulatory status for each patient [9].

The understanding of cerebral autoregulation is especially important in ICH since blood pressure management is the mainstay of current treatment options [10]. With impaired cerebral autoregulation, aggressive lowering of blood pressure may lead to undesirable cerebral ischemia; but maintaining high blood pressure may theoretically lead to hyperemia and hematoma expansion. Hence, adjusting blood pressure with the understanding of cerebral autoregulation status may be more effective in maintaining optimal cerebral blood flow, which could potentially lead to better outcomes. Although studies using various neuroimaging techniques suggest that "static" cerebral autoregulation in the peri-hematoma region is likely preserved after ICH [11-13], there are limited data on "dynamic" cerebral autoregulation after ICH [14]. Thus, we conducted a case-control study to compare dynamic cerebral autoregulation capacity between patients with acute ICH and community-dwelling healthy subjects.

### Static vs. Dynamic Cerebral Autoregulation

Most of the early studies of cerebral autoregulation were assessed by gradually raising or lowering blood pressure and measuring the cerebral blood flow (CBF) responses to determine if steady-state CBF values have changed [1,15]. This method reflects *static *cerebral autoregulation and captures the steady-state relationship between gradual changes in pressure and flow [16]. Most of the neuroimaging studies assessing the changes in CBF or oxygen extraction fraction (OEF) with changes in blood pressure reflect the "static" component of cerebral autoregulation. In contrast, *dynamic *cerebral autoregulation measurements assess the "robustness" of dynamic cerebral vessel caliber changes in response to sudden changes in blood pressure, which cannot be assessed with most static imaging techniques.

## Methods

### TCD Ultrasound and Dynamic Cerebral Autoregulation

TCD ultrasound is a noninvasive and readily available diagnostic tool that can be used to assess dynamic cerebral autoregulation. Recent developments in Doppler technology have enabled researchers to measure spontaneous beat-to-beat changes in mean blood flow velocity (MFV) of the major cerebral arteries in response to spontaneous mean arterial pressure (MAP) oscillations, allowing investigators to quantify the dynamic relationship between MFV and MAP without the substantial risk of manipulating blood pressure [17]. This allows assessment of "dynamic" cerebral autoregulation by analyzing the beat-to-beat spontaneous oscillations of MFV and MAP [18]. Dynamic cerebral autoregulation can be determined by the transfer function gains and phase shift angles of the two oscillating signals. In the cerebral circulation, the gain of the blood pressure-flow transfer function quantifies the damping effect of cerebral autoregulation on the magnitude of blood pressure oscillations. A low gain value indicates intact dynamic cerebral autoregulation, whereas a high gain value can be interpreted as impaired dynamic cerebral autoregulation [18,19]. The phase shift represents the temporal difference between blood flow oscillations with respect to blood pressure oscillations and is considered a surrogate measures for the time delay of the autoregulatory response. If oscillations of cerebral blood flow and blood pressure are synchronous (i.e., phase shift = 0 degrees), then they are in phase and indicate impaired dynamic cerebral autoregulation, whereas phase shifts approaching 90 degrees indicate more efficient dynamic cerebral autoregulation [18,20]. Because dynamic cerebral autoregulation is a relatively slow process and can take 2-10 seconds to engage [17], frequency-domain analysis of dynamic autoregulation is typically studied at frequency ranges < 0.15 Hz [21].

### Subjects

The study received approval from the Brigham and Women's Hospital institutional ethical standards committee on human experimentation for any experiments using human subjects. A written consent was obtained from the subjects or their healthcare proxies.

### ICH Patients

From August 2007 to June 2009, we prospectively screened 37 patients admitted to the neurointensive care unit at Brigham and Women's Hospital with diagnosis of spontaneous ICH. Inclusion criteria were age greater than 18, evidence of acute ICH on admission by computed tomography (CT), symptom onset within 72 hours from the study and hemodynamic stability. Exclusion criteria included traumatic hemorrhage, history of chronic obstructive lung disease, unstable blood pressure requiring vasopressor agents, other central neurological co-morbidities (e.g. central nervous system vasculitis, multiple sclerosis, sickle cell disease, or tumor) and inadequate TCD windows. All head CT scans were reviewed independently by one of the study investigators (K.N.) using a standardized protocol, blinded to cerebral hemodynamic data, and hematoma volume was calculated from the most recent brain CT prior to the TCD study by using the "ABC/2" volume method [22]. The location and etiology of ICH were determined by one of the investigators (K.N.) based on clinical history and CT findings. Because our TCD method was intended to assess cerebral autoregulation capacity primarily in the middle cerebral artery (MCA) distribution, we excluded patients with thalamic (4 patients), pontine (2 patients) and primary intraventricular hemorrhage (1 patient) in the final analysis. We also excluded 1 patient with hemorrhage related to arteriovenous malformation (AVM) because AVM's unique flow dynamic may affect cerebral autoregulation. Three patients were excluded because time-to-study exceeded 72 hours from the symptom onset. A total of 21 patients with lobar or basal ganglia ICH with adequate TCD window for MCA insonation were included in the study.

### Control Group

Twenty-three age-matched healthy subjects (65 ± 9 years) with no history of stroke or major vascular risk factors were drawn from a group of control subjects involved in another study [23]. In the control group, only the right MCA MFV was measured and used for comparative analysis.

### Study Procedures

Upon arrival to the emergency department, each patient underwent baseline brain CT and evaluation by a neurologist. The subjects were placed in a 30-degree recumbent position on a standard hospital bed. They were instrumented for measurement of heart rate and non-invasive beat-to-beat arterial blood pressure monitoring (Finapres, Ohmeda Monitoring Systems, Englewood, CO) as previously described [24]. TCD ultrasonography (Multi Dop X4, DWL-Transcranial Doppler Systems Inc., Sterling, VA) was used to measure changes in MCA MFV. The MCA signals were identified on ipsilateral and contralateral sides (hemorrhagic and nonhemorrhagic hemispheres) according to the criteria of Aaslid et al. [25] and recorded simultaneously for 5 minutes. A Spencer probe fixation device was used to stabilize the Doppler probes for the duration of the study. The envelope of the velocity waveform, derived from a fast Fourier analysis of the Doppler frequency signal, was digitized at 500 Hz, displayed simultaneously with the blood pressure waveform and stored for later off-line analysis. In the control group, MCA MFV and blood pressure were recorded in the supine position using a similar technique.

### Data Analysis

All data were displayed and digitized in real time at 500 Hz with commercially available data acquisition software (Windaq, Dataq Instruments). The beat-to-beat cycles were determined by detecting the peak of each arterial pressure waveform and flow velocity waveform. The MAP and MFV values were determined from the integrals of each waveform using a custom Matlab program to calculate the systolic and diastolic blood flow velocity from peak and minimum TCD values within each beat cycle. Cerebrovascular resistance index (CVR_i_) was calculated as the ratio of MAP to MFV. Heart rate was determined from peak to peak times. All data segments for MAP and MFV were visually inspected and edited for artifact and ectopy, and only steady-state data were used for analysis.

A power spectrum analysis technique based on the Welch algorithm of averaging periodograms was used. The 5-minute time series were interpolated at 100 Hz to obtain equidistant time intervals. For fast Fourier analysis a Hanning window of 30 seconds was used with two-third overlap and transformed to its frequency representation squared. The periodograms were averaged to produce the spectrum estimate. Coherence between MAP and MFV were calculated from the cross-spectra and autospectra of steady-state data segments as follows: (cross-spectra)^2^/(input signal autospectrum) × (output signal autospectrum). Transfer functions were determined by dividing the cross-spectrum by the input autospectrum for each vessel insonated [19]. With a custom MATLAB program, transfer gains and phases were calculated for each subject using the MAP and normalized MFV signal autospectra (in percentage change in cm/s/mm Hg) over the two specified frequency ranges: low (0.03-0.15 Hz) and high (0.15-0.5 Hz). The MCA MFV was normalized to account for inter-subject variability [4].

### Statistical Analysis

Data were analyzed using commercially available statistical software (SPSS 18.0, Chicago, IL). Descriptive statistics were used to describe patient characteristics, clinical data and hematoma sizes. Inter-group differences were tested using *χ^2 ^*test for categorical data and *t*-test for normally distributed continuous variables. Within the ICH group, intra-individual differences in MFV, transfer function coherence, gains, and phases between the ipsilateral and contralateral sides were tested using a repeated measures two-way ANOVA using hematoma size as a covariate. Relationships between hematoma size and coherence, gains and phases were tested using Pearson correlation analysis. Data are presented as mean ± SD, and levels of *p *< 0.05 are considered statistically significant.

## Results

Clinical and hemodynamic characteristics are shown (Table 1). Mean time from symptom onset to the TCD measurement in the ICH group was 34 ± 14 hours. Eight patients (38%) had hypertensive hemorrhage, 4 patients (19%) had warfarin-related hemorrhage, 2 patients (10%) had hemorrhage due to probable cerebral amyloid angiopathy (CAA), 1 patient (5%) had hemorrhage related to venous sinus thrombosis, and 4 patients (19%) had hemorrhage of undetermined etiology. The mean hematoma volume was 36 ± 27 cm^3^. None of the subjects required surgical evacuation of the space-occupying hematoma. The ICH group demonstrated lower MCA MFV on both sides and higher CVR_i _compared to controls (Table 1).

**Table 1:** Clinical and Hemodynamic Characteristics

	ICH	Control	*P *value
***N***	21	23	
**Age, yr**	66 ± 15	65 ± 9	0.80
**Gender (male:female)**	8:13	8:15	0.82
**ICH Size, cm^3^**	36 ± 27	NA	
**Diabetes mellitus, *n *(%)**	4 (19)	NA	
**Hypertension, *n *(%)**	12 (57)	NA	
**Secondary IVH, *n *(%)**	7 (33)	NA	
**Location of hematoma, *n *(%)**		NA	
**Lobar**	13 (62)		
**Basal Ganglia**	8 (38)		
**Time to TCD, hr**	34 ± 14	NA	
**SBP, mmHg**	130 ± 26	134 ± 26	0.59
**DBP, mmHg**	53 ± 13	65 ± 19	0.02
**MAP, mmHg**	77 ± 15	89 ± 21	0.04
**Heart rate, beat/min**	74 ± 11	63 ± 14	0.005
**MCA MFV, cm/s**			
**Ipsilateral**	49.8 ± 22.5	66.6 ± 18.9	0.01
**Contralateral**	50.6 ± 24.2		0.02
**MCA CVR_i_, mmHg·s·cm^-1^**			
**Ipsilateral**	1.91 ± 1.01	1.42 ± 0.45	0.04
**Contralateral**	2.01 ± 1.24		0.04

**Figure 1:**
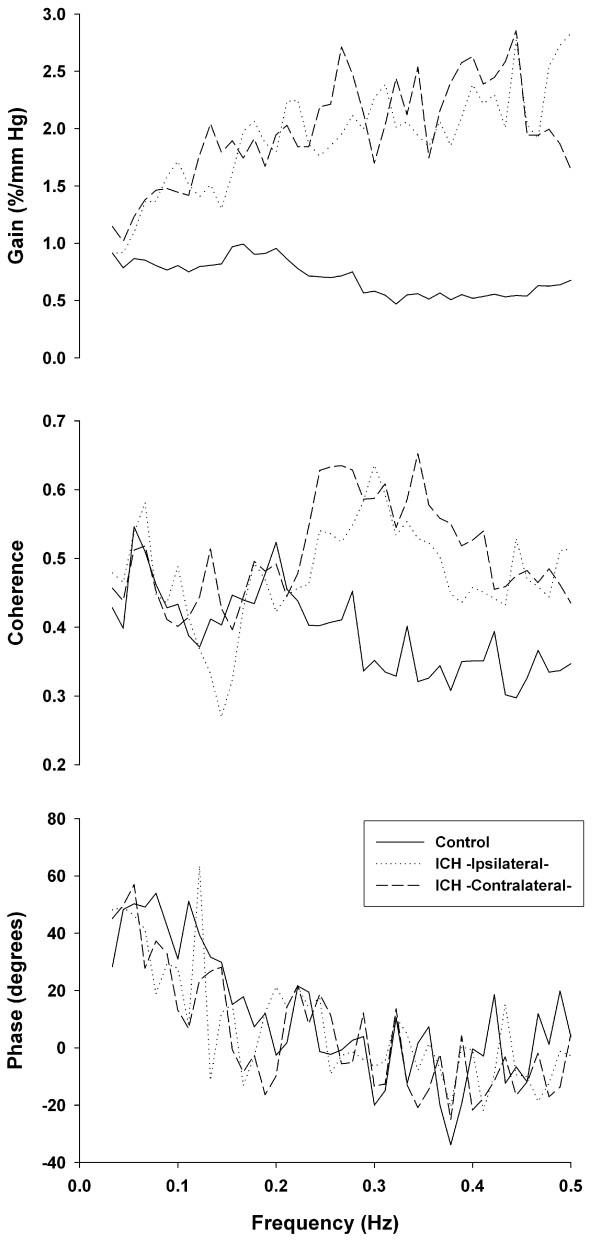
**Cross-spectral function analysis for the ICH group and the control**. Cross-spectral function analyses of the beat-to-beat mean arterial pressure and middle cerebral artery mean flow velocity in the 0.03-0.5 Hz ranges were used to determine the gain, coherence and phase values for the ICH group (ipsilateral and contralateral sides) and the control group.

**Figure 2:**
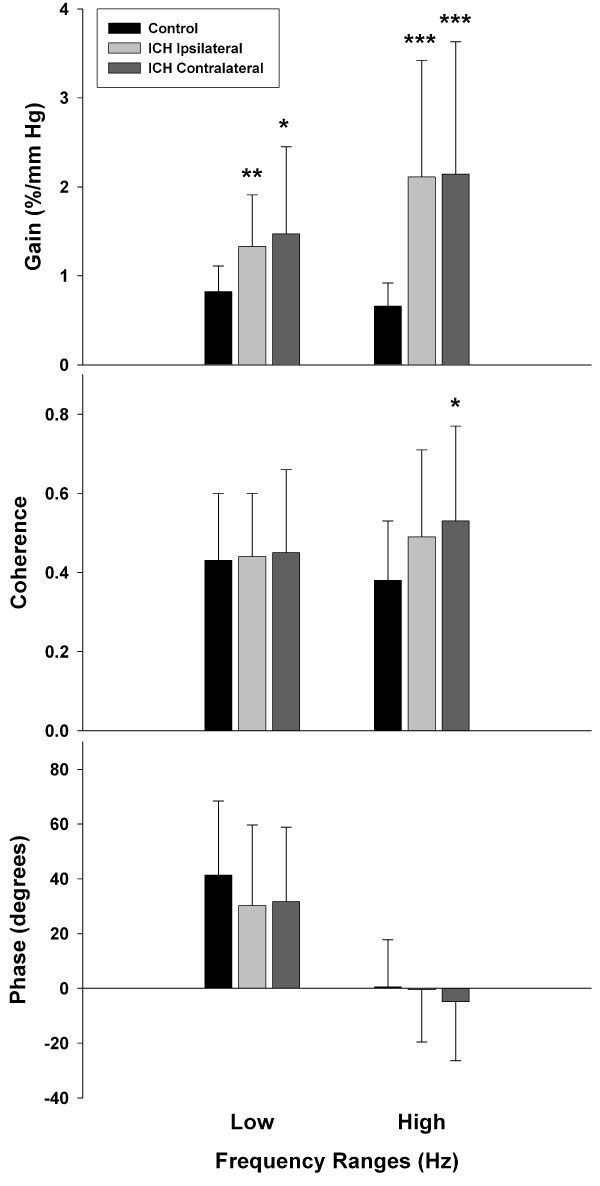
**Transfer function analysis for the ICH group and controls**. Comparison of transfer function gain, coherence, and phase in the low (0.03-0.15 Hz) and high (0.15-0.5 Hz) frequency ranges between the ICH group (n = 21) and controls (n = 23). Values are expressed as mean (bar) ± SD (error bar). Ipsilateral and contralateral sides of the ICH group were compared to controls. Significant differences between the two groups are shown. *p < 0.01, **p < 0.001, ***p < 0.0001.

Figure 1 presents the results of the cross-spectral function analysis for the ICH group and the control. Overall, the transfer function gains for the ICH group were greater than that for the control for the majority of harmonics in the frequency range 0.03-0.5 Hz. When grouped into two frequency ranges: low (0.03-0.15 Hz) and high (0.15-0.5 Hz), the ICH group showed higher gains than the control in the low frequency range (ipsilateral: 1.33 ± 0.58%/mmHg, *p *= 0.0005; contralateral: 1.47 ± 0.98%/mmHg, *p *= 0.004; vs. control: 0.82 ± 0.30%/mmHg) and high frequency range (ipsilateral: 2.11 ± 1.31%/mmHg, *p *< 0.0001; contralateral: 2.14 ± 1.49%/mmHg, *p *< 0.0001; vs. control: 0.66 ± 0.26%/mmHg) (Figure 2). Furthermore, the ICH group also had higher coherence in the contralateral hemisphere than the control (ICH contralateral: 0.53 ± 0.38 vs. control: 0.38 ± 0.15, *p *= 0.02;) in the high frequency range (Figure 2).

**Figure 3:**
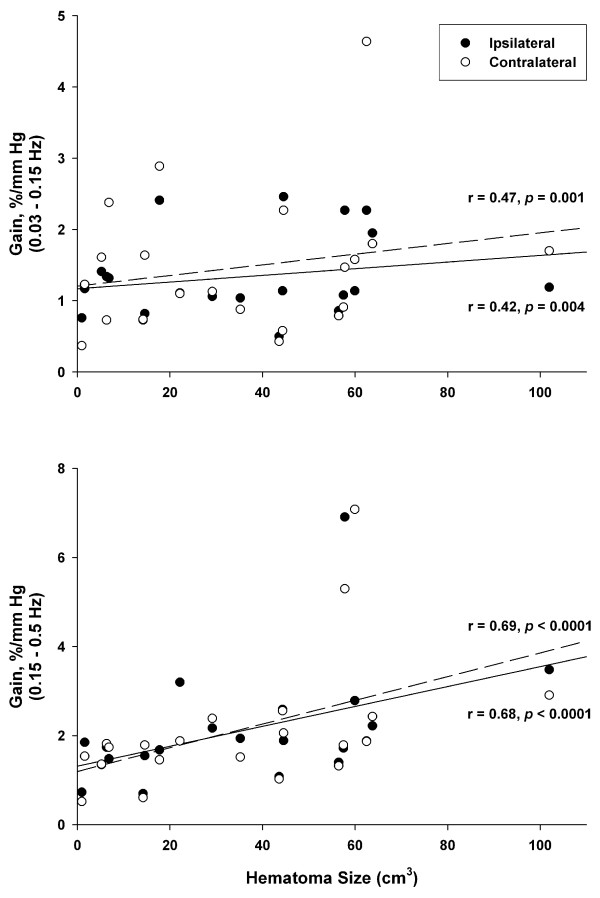
**Relationship between hematoma size and transfer function gain in the ICH group**. Within the ICH group (n = 21), correlation between hematoma size and transfer function gain at 0.03-0.15 Hz and 0.15-0.5 Hz frequency ranges are shown. Lines represent linear regression for ipsilateral (dashed lines) and contralateral (solid lines) hemisphere.

Within the ICH group, comparison of MFV, CVR_i_, coherence, gains, and phases between the ipsilateral and contralateral sides showed no significant differences. Pearson correlation analyses showed significant correlation between hematoma size and gains in the low and high frequency ranges in both hemisphere (Figure 3).

## Discussion

Our study shows that compared to healthy controls, patients with lobar or basal ganglia ICH have higher MCA transfer gains in both hemispheres involving a wide range of frequencies. These findings suggest that dynamic cerebral autoregulation is less effective in ICH patients. Dynamic cerebral autoregulation is a relatively slow-responding process and thought to primarily operate in the lower frequency ranges (i.e. < 0.15 Hz). The increased gain seen in the low frequency range in our study supports the concept that slow-responding dynamic cerebral autoregulation is less effective in the ICH patients.

Interestingly, gains in the high frequency range (0.15-0.5 Hz), a frequency range that is considered a much faster response (2-7 seconds) than what is generally considered for dynamic cerebral autoregulation, were also greater in the ICH group. Panerai et al. have shown similar results in a group of neonates where those with normal cerebral autoregulation had lower transfer gain in the 0.25-0.43 Hz range (> 0.15 Hz range) compared to those with impaired cerebral autoregulation [26]. Although it is possible that the differences in gain seen in this high frequency may reflect systemic hemodynamic factors seen in neurocritical care patients compared to the controls, we speculate that it may also reflect the faster myogenic response of the small vessels. The high frequency gain has been correlated with CO_2 _reactivity and also inversely correlated with the degree of proximal artery stenosis, [27] and thus may reflect a fast vasomotor response. However, the clinical importance of transfer gain values in this high frequency range is uncertain, and its relationship to the mechanism of dynamic cerebral autoregulation needs to be further investigated.

Our findings are also consistent with the study by Schramm et al. [14] which showed that dynamic cerebral autoregulation of both hemispheres were impaired after acute brain injury from day 1 to 4 with a nadir on day 4. Compared to their study, which was limited by the small sample size and mixed cases (4 ICH and 12 TBI patients), we were able to show similar findings with the non-traumatic ICH patients with a greater number of patients. Furthermore, the significant correlation between hematoma size and gains on both hemispheres in our study also supports the idea that the presence of hematoma may have more direct impact on dynamic autoregulation. Interestingly, these correlations between hematoma size and gains were more prominent in the high frequency. We speculate that large hematomas are associated with higher intracranial pressure and lower intracranial compliance compared to smaller hematomas. With lower compliance, the affected area of the brain would be "stiffer" and may impair the fast-responding myogenic response. Another possible explanation is that with higher intracranial pressure, the cerebral perfusion pressure would likely be reduced, which may also impair cerebral autoregulation. These explanations would be consistent with the findings of Panerai et al. [28] who showed that in severe head injury patients, the high ICP group had greater deterioration of dynamic cerebral autoregulation than the low ICP group. Unfortunately, we did not have ICP measurements on our patients so we could not examine the relationship between ICP and blood flow in these patients.

Since our measurements were made post ictus and we do not have any pre ICH measures, we can not rule out the possibility that dynamic cerebral autoregulation was already impaired in the ICH group prior to hemorrhage due to underlying vascular risk factors such as hypertension. In fact, the *static *cerebral autoregulation curve is thought to be shifted to the right in people with untreated hypertension [29]. However, the *dynamic *cerebral autoregulation has been shown to be preserved with those with controlled or uncontrolled hypertension [19]. Therefore, it is unlikely that our findings of bilateral impairment of dynamic cerebral autoregulation in ICH patients are explained by their long-standing history of hypertension.

Critical for the interpretation of our data is to understand the extent to which the differences in MFV and CVR_i _between the two groups may have contributed to our observed differences in transfer gains. We speculate that antihypertensive and sedative medications administered to the ICH group as part of standard protocol [10] likely resulted in the lower blood pressure and lower MCA MFV seen in our ICH patients. Although it is possible that these medications may have altered dynamic cerebral autoregulation, we believe that these effects were minimal because most of the patients received intravenous labetalol for acute antihypertensive treatment and intravenous propofol for sedation, neither of which has been shown to impair cerebral autoregulation [30,31]. The ICH group had lower MFV and higher CVR_i _compared to controls, suggesting that the small arteries and arterioles were likely more vasoconstricted in the ICH group. Since many studies suggest more effective cerebral autoregulation with vasoconstriction [19,32,33]; this could not explain why our ICH patients with higher CVR_i _(more vasoconstriction) had higher transfer gains than controls. This further supports the hypothesis that compared to healthy controls, dynamic cerebral autoregulation of the ICH patients is intrinsically more impaired, independent of the effect of vessel caliber.

Another important factor to note is the potential effect of blood pressure variability on transfer gain. Theoretically, if patients have significantly higher blood pressure variability, it may be more difficult for even intact autoregulation to compensate for this variability. However, our ICH patients had lower blood pressure variability than the control group, and thus the higher gain seen in the ICH patients suggests that even with reduced blood pressure changes, autoregulation was still unable to compensate effectively.

To date, there is no intervention that significantly improves clinical outcome after spontaneous ICH. Several clinical trials are currently ongoing to test whether aggressive blood pressure lowering prevents hematoma expansion and improves clinical outcome [34-36]. However, none of the studies are addressing cerebral autoregulation as a potential contributing factor for hematoma expansion and clinical outcome. Recent studies suggest that cerebral autoregulation status is associated with clinical outcome after spontaneous ICH [8,37]. Specifically, a study by Reinhard et al. has shown that ICH patients who had cerebral autoregulation deterioration between day 3 and 5 were associated with poor 3-month functional outcome [37]. Although it is uncertain whether subacute deterioration in autoregulation merely reflects the severity of the disease or whether it contributes to secondary neuronal injury and poor outcome, these studies support the idea that frequent monitoring of cerebral autoregulation status may be needed in selected ICH patients.

### Study Limitations

This was an exploratory case-control study to assess the differences in dynamic cerebral autoregulation between patients with acute spontaneous ICH and healthy controls. The lack of a separate control group with similar vascular risk factors is the major limitation of the study since we were unable to directly address whether our observation was due to their underlying chronic vasculopathy or due to acute deterioration as a result of acute ICH. Furthermore, since the patients treated in the intensive care unit may have other unaccounted factors not present in the control group that could affect the results of transfer function analysis, our results should be interpreted with caution. Although we excluded subjects with chronic obstructive lung disease, and none of the patients had oxygenation or ventilation problems during the study, alterations in PaCO_2 _or end-tidal CO_2 _could have introduced variations in the vasomotor reactivity and cerebral blood flow between patients. Our study was conducted over a range of 72 hours from the ictus, which may have combined the hyperacute and early subacute stages of ICH, which may have differed. Given the dynamic nature of neurocritical care patients who may have fluctuating hemodynamic and autoregulatory status, one cerebral autoregulation measurement from a 5 minute recording may not be sufficient to capture their overall cerebral autoregulatory status.

## Conclusions

In conclusion, our study indicates that dynamic cerebral autoregulation may be less effective in patients with spontaneous lobar or basal ganglia ICH compared to healthy controls. Our findings also suggest that dynamic cerebral autoregulation may be further impaired in patients with larger hematomas; however, a larger prospective study is needed to confirm this observation.

## Competing interests

The authors declare that they have no competing interests.

## Authors' contributions

KN participated in the conception and design of the study, performed the TCD measurements on the ICH patients, conducted the statistical analyses, and was responsible for drafting and finalizing the manuscript. JMS performed the TCD measurements on the control group, conducted the Matlab analyses of the data, helped in the interpretation of the data, and helped to draft and finalize the manuscript. SLL participated in patient recruitment and coordination of the study, participated in the TCD measurements of the ICH patients, and helped to draft and finalize the manuscript. FAS participated in the conception and design of the study, analyses and interpretation of the data, and helped to draft and finalize the manuscript. All authors read and approved the final manuscript.

## Pre-publication history

The pre-publication history for this paper can be accessed here:

http://www.biomedcentral.com/1471-2377/11/108/prepub
